# Tissue and Time Optimization for Real-Time Detection of Apple Mosaic Virus and Apple Necrotic Mosaic Virus Associated with Mosaic Disease of Apple (*Malus domestica*)

**DOI:** 10.3390/v15030795

**Published:** 2023-03-21

**Authors:** Sajad Un Nabi, Javid Iqbal Mir, Salwee Yasmin, Ambreena Din, Wasim H. Raja, G. S. Madhu, Shugufta Parveen, Sheikh Mansoor, Yong Suk Chung, Om Chand Sharma, Muneer Ahmad Sheikh, Fahad A. Al-Misned, Hamed A. El-Serehy

**Affiliations:** 1 ICAR-Central Institute of Temperate Horticulture, Srinagar 191132, Jammu & Kashmir, India; 2 ICAR-Indian Institute of Horticultural Research, RS-Chettalli, Bangaluru 571248, Karnataka, India; 3 Department of Plant Resources and Environment, Jeju National University, Jeju-si 63243, Republic of Korea; 4 Department of Zoology, College of Science, King Saud University, Riyadh 11451, Saudi Arabia

**Keywords:** apple, mosaic, virus, detection, spatial, temporal, expression

## Abstract

Besides apple mosaic virus (ApMV), apple necrotic mosaic virus (ApNMV) has also been found to be associated with apple mosaic disease. Both viruses are unevenly distributed throughout the plant and their titer decreases variably with high temperatures, hence requiring proper tissue and time for early and real-time detection within plants. The present study was carried out to understand the distribution and titer of ApMV and ApNMV in apple trees from different plant parts (spatial) during different seasons (temporal) for the optimization of tissue and time for their timely detection. The Reverse Transcription-Polymerase Chain Reaction (RT-PCR) and Reverse Transcription-quantitative Polymerase Chain Reaction (RT-qPCR) was carried out to detect and quantify both viruses in the various plant parts of apple trees during different seasons. Depending on the availability of tissue, both ApMV and ApNMV were detected in all the plant parts during the spring season using RT-PCR. During the summer, both viruses were detected only in seeds and fruits, whereas they were detected in leaves and pedicel during the autumn season. The RT-qPCR results showed that during the spring, the ApMV and ApNMV expression was higher in leaves, whereas in the summer and autumn, the titer was mostly detected in seeds and leaves, respectively. The leaves in the spring and autumn seasons and the seeds in the summer season can be used as detection tissues through RT-PCR for early and rapid detection of ApMV and ApNMV. This study was validated on 7 cultivars of apples infected with both viruses. This will help to accurately sample and index the planting material well ahead of time, which will aid in the production of virus-free, quality planting material.

## 1. Introduction

Apple (*Malus domestica* L.) belongs to the family *Rosaceae* and is being widely grown across the temperate regions of the world as well as India [1,2]. Viral diseases pose a real challenge to apple cultivation, including reductions in plant vigor, delays in fruit ripening, graft incompatibility issues or overall loss in yields both quantitatively and qualitatively [3,4]. Viruses may also remain latent, causing plant to grow slowly with a reduced lifespan, but these effects may go unnoticed unless crops are visibly damaged [5,6]. Among viral diseases, apple mosaic disease (AMD) is the most economically important viral disease associated with apple cultivation, having a widespread distribution and representing a major threat to the apple industry throughout the world [7,8]. The mosaic symptom pattern on the leaves has a direct bearing on the photosynthetic ability of the plant, reducing it up to 46% and thereby reducing the fruit yield by 20–30% [4,9]. In a previous study, only the apple mosaic virus (ApMV) was found to be associated with AMD [10]. However, several other viruses, viz., such as the apple necrotic mosaic virus (ApNMV), prunus necrotic ring spot virus (PNRSV) and cucumber mosaic virus (CMV) [11,12,13], have also been associated with AMD. Earlier research based on immunological and molecular methods (DAS-ELISA and RT-PCR) found ApMV to be the only causal agent associated with AMD in India from Jammu and Kashmir and Himachal Pradesh [14,15,16,17]. However, the association of both ApMV and ApNMV with mosaic disease in several apple cultivars from India has been reported [18]. Due to the non-availability of chemical viricides, it is difficult to control viral pathogens. Hence, the only effective way to prevent viral spread in perennial plants is through the use of virus-free planting material via indexing of scion wood and rootstocks using robust, timely and precise detection methods [19,20]. Diagnostic assays play an important role in the detection of plant viruses through their spatial and temporal distribution or selected expression in symptomatic, as well as asymptomatic, host plants [21,22]. Although DAS-ELISA and symptomatic visualization can help in the detection of viruses, a low titer of virus combined with a limitation to certain time period in the growing season can give false results. To avoid such ambiguity, reverse transcription-polymerase chain reaction (RT-PCR) can provide an alternate route [19]. The technique is sensitive even during seasons of low virus titer and reproducible with limited risk of contamination compared to end-point RT-PCR [23,24]. Most importantly, both viruses (ApMV or APNMV) belong to the genus *Illarvirus* and being labile in nature, show uneven distribution in apple trees and decreases variably with temperature [25,26]. Hence, proper time and proper tissue is needed for the detection of both viruses to avoid detection despite being present in the plant. The objective of the present study was to detect and quantify the titer of both ApMV and ApNMV from different parts of plant (spatial) during different seasons (temporal) of a year from two mosaic-infected cultivars (Oregon Spur, OS and Golden Delicious, GD) for the optimization of tissue and time for the real-time detection of both viruses.

## 2. Materials and Methods

### 2.1. Field Sampling for Tissue Selection and Processing

**Figure 1:**
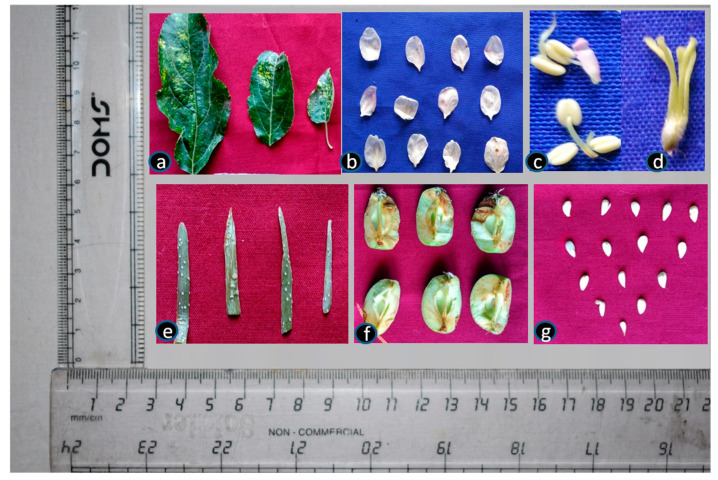
Different plant parts/tissues with presence or absence of symptoms noted for virus detection and quantification. (**a**) Symptomatic leaf and pedicel, (**b**) petals, (**c**) anther, (**d**) flowers without petal, (**e**) bark, (**f**) fruit and (**g**) seed.

The experiment was conducted using plants from cultivars of Golden Delicious (GD) and Oregon Spur (OS) established at the field gene bank of ICAR-Central Institute of Temperate Horticulture (CITH), Srinagar, India. Both cultivars selected were of the same age (10 years), from the same rootstock of Malling Merton (MM) 106 (M.2 × Northern Spy) and grown under similar agronomic practices. The cultivar GD is used as a pollinizer, whereas the cultivar OS is being commercially cultivated in apple growing regions of India. The symptomatic plants showing viral symptoms and asymptomatic plants were evaluated during all three seasons of year, i.e., the spring, summer and autumn season. Collections included: leaf, flower, petal, bark and pedicel during the spring season (April) of 2021–2022; leaf, bark, pedicel, immature fruit and immature seed in the summer season (July); and leaf, pedicel, bark, mature fruit and mature seeds in the autumn season (October) (Figure 1). A total of 42 samples were collected from both cultivars in which 5 samples represented each tissue in each cultivar (3 samples × 7 tissues × 2 cultivars = 42). The tissue samples of 50–80 gm from asymptomatic and symptomatic designated plants of the GD and OS cultivars were collected in liquid nitrogen for the detection and quantification of ApMV and ApNMV. The samples were stored at −20 °C and conventional RT-PCR and real-time RT-PCR analysis were used to study the distribution and quantification assays, respectively, for ApMV and ApNMV in different tissues.

### 2.2. Total Ribonucleic Acid (RNA) Extraction and cDNA Synthesis 

Total RNA was extracted from 100 mg of each tissue using plant total RNA Mini Kit (Qiagen RNeasy kit) as per the manufacturer’s protocol with slight modifications. The integrity of RNA was ascertained on 1% diethylpyrocarbonate (DEPC)-treated agarose gel, followed by quantitative and qualitative analysis to ascertain the purity on Nanodrop (Thermo scientific, Mumbai, India). The RNA was stored at −80 °C for further use. The coat protein (CP) gene-specific complementary deoxyribonucleic acid (cDNA) for ApMV and ApNMV was prepared using RevertAid first strand cDNA synthesis kit (Thermo scientific K1622) according to the manufacturer’s protocol. This was accomplished using 2 µg of RNA in a 20 µL mixture containing 1X first strand buffer, 0.5 µM dNTPs (Deoxynucleoside triphosphates), 0.5 uM of each primer, Revert Aid Reverse Transcriptase (200 U) and Ribolock RNase Inhibitor (20 U).The reaction was performed in a thermal cycler (Takara, Japan) at 42 °C for 60 min.

### 2.3. Reverse Transcriptase-PCR (RT-PCR)

The master mix for PCR amplification was prepared using synthesized cDNA (2 µL) as a template in a 20 µL reaction volume containing 1Xtaq buffer, 0.5 mM dNTPs, 1 U of Taq polymerase (HIMEDIA), 0.25 µM of each primer of APMV (F- 5′ATCCGAGTGAACAGTCTATCCCTC3′, R-5′GTAACTCACTCGTTATCACGTAC 3′) and ApNMV (F-5′ATGGTGTGCAATCGCTGTCAANMV3′, R- 5′CATCGACCATAAGGATATCA3′) and 1.5 mM MgCl_2_ (magnesium chloride).The program was set up for 35 cycles with denaturation at 94 °C for 30 s, annealing at 46 °C (ApNMV) and 53 °C (ApMV) for 40 s, followed by extension at 72 °C for 30 s and a final elongation step at 72 °C for 10 min. Amplified products were visualized after electrophoresis in ethidium bromide (EtBr)-stained 1.2% agarose gel and the amplicon size was estimated using a 100 bp/1 kb DNA ladder (Invitrogen).

### 2.4. Quantitative Real-Time PCR Analysis (qRT-PCR)

To determine the virus titer in different tissue samples of GS and OS cultivars, quantitative gene expression was carried out in RT-qPCR using SYBR green I Master mix kit (Himedia, MBT074). Data from the reactions were analyzed by an amplification plot to determine the variable threshold cycle (Ct), defined as the cycle at which a significant increase in fluorescence occurs. The total reaction volume was 20 µL, which included 2 µL cDNA, 10 µL SYBR Green, 1 µL of each of 10 µM forward and reverse coat protein primer for ApMV and ApNMV and 6 µL PCR grade water. The pre-incubation was carried out at 95 °C for 5 min, followed by 35 cycles at 95 °C for 20 s, 56 °C and 46 °C for ApMV and ApNMV, respectively, for 15 s and 72 °C for 15 s. The tubulin gene was used as the reference gene for both primers. For relative quantification, the positive calibrator was taken as CP gene expression in leaves during the spring and autumn, whereas it was in seeds during the summer. The Ct values of the positive calibrator/control and the samples were normalized to the endogenous housekeeping gene tubulin. Relative gene expressions were determined according to the ∆∆Ct (relative fold gene expression) method using the formula described in [27]. 

2^−∆∆Ct^, where ∆∆Ct = [∆] Ct sample − [∆] Ct reference

[∆] Ct sample—Ct value for any sample normalized to the endogenous housekeeping gene

[∆] Ct reference—Ct value for the reference sample normalized to the endogenous housekeeping gene

### 2.5. Validation of Tissue and Time

Seven apple cultivars (OS, Welson Red June-WRJ, GD, Sharp Earliest-SE, Gala Mast-GM, June Eating-JE and Ambri-A) were selected for the validation of tissue and time optimization for real-time detection of ApNMV/ApMV. The optimized tissues were collected from seven mosaic-infected apple cultivars maintained at the gene bank of ICAR-CITH Srinagar during three seasons in 2021–2022. Nearly 200 samples were collected from seven cultivars in which 5 samples represented each standardized tissue in each cultivar to validate the results.

### 2.6. Data Analysis

The experiment was conducted under a completely randomized design (CRD) and the data was analyzed through an analysis of variance test (ANOVA) using SAS package. All the measurements with *p* < 0.01 were assumed to be statistically significant. For relative gene expression studies, all the experiments were carried out in triplicates i.e., one sample of the same tissue per replicate. 

## 3. Results

### 3.1. Symptomatology of Mosaic Disease on Different Plant Parts

**Figure 2:**
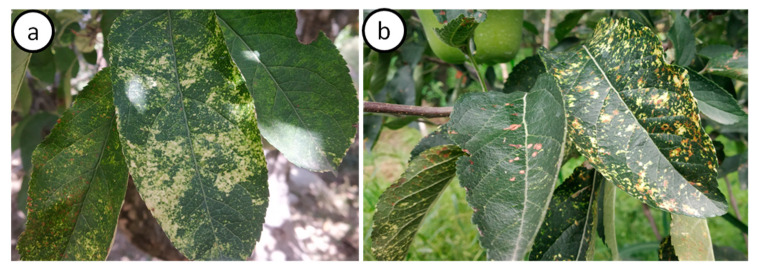
The symptoms of mosaic and necrotic mosaic on two cultivars: Golden Delicious (**a**) and Oregon Spur (**b**).

The symptoms such as chlorosis, mosaic and necrotic spots were observed on leaves of cultivar OS, whereas only mosaic was observed on cultivar GD (Figure 2). Mosaic or mosaic-necrosis symptoms ranged from small pale-yellow spots scattered across an entire leaf or part of a leaf to large, contiguous chlorotic spots covering an entire leaf lamina, along with necrosis on some cultivars (OS). Neither mosaic nor necrosis was observed on different floral parts, fruits or seeds in both cultivars. Interestingly, the symptoms of mosaic or mosaic-necrosis were distinct during the spring season; however, in the summer and autumn season, the symptoms of mosaic were mostly masked and less prominent.

### 3.2. Detection of ApMV and ApNMV Using RT-PCR

Both of the viruses, ApMV and ApNMV, were detected in all the tissues during different seasons, depending on the tissues available in each season. During the summer season, the RT-PCR couldn’t detect both viruses in leaves.

#### 3.2.1. Detection of ApMV

**Figure 3:**
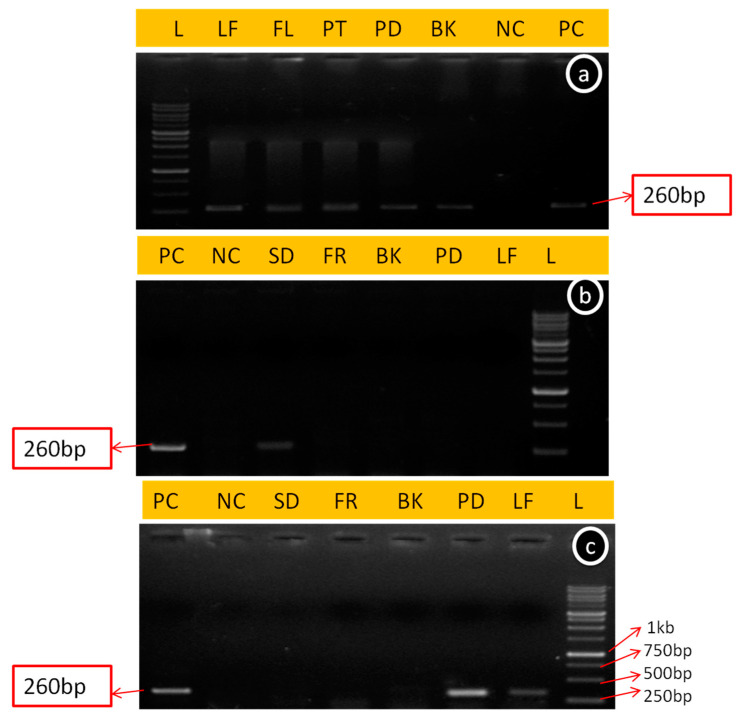
Amplicon of 260 bp amplified from ApMV-infected tissues of cultivar Golden Delicious (GD) plant tissues by conventional RT-PCR during the spring season (**a**), summer season (**b**) and autumn season (**c**), where L: ladder 100 bp, LF: Leaf, FL: Flowers, GD4:Bark, PT: Petals, PD: Pedicel, BK: Bark, FR: Fruit, SD: Seed, NC: Negative control, and PC: Positive control.

The ApMV was tested in all the tissue samples (anther, leaf, pedicel, petal, flowers without petals, bark, fruit and seed) through conventional RT-PCR during all three seasons (spring, summer and autumn). It was observed that the specific primer of coat protein gene amplified 252 bp, corresponding to ApMV in tested samples (depends on their availability during different seasons) except in healthy controls in both cultivars. The amplified products of ApMV from different tissues, along with the positive control, are shown in Figure 3. The presence of ApMV in different tissues during different seasons in OS and GD cultivars was the same as that presented in Table 1.

#### 3.2.2. Detection of ApNMV

**Figure 4:**
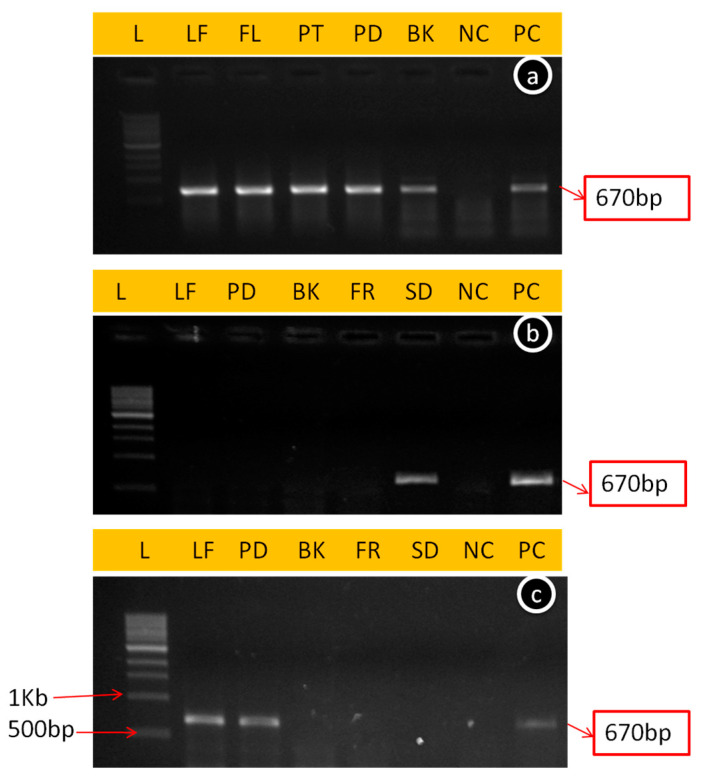
Amplicon of 670 bp amplified from ApNMV-infected tissues of cultivar Golden Delicious (GD) plant tissues by conventional RT-PCR during the spring season (**a**), summer season (**b**) and autumn season (**c**), where L: ladder 1 kb, LF: Leaf, FL: Flowers, GD4:Bark, PT: Petals, PD: Pedicel, BK: Bark, FR: Fruit, SD: Seed, NC: Negative control, and PC: Positive control.

The ApNMV was also tested in all tissue samples through conventional RT-PCR during all three seasons. It was observed that the specific primer of the coat protein gene of ApNMV amplified 670 bp (Figure 4) in tested samples (depending on their availability during different seasons) except in healthy control in both cultivars. The presence of ApNMV in different tissues during different seasons in both cultivars was the same as that presented in Table 1.

### 3.3. Relative Quantification of ApMV and ApNMV through Real-Time PCR in Different Tissues during Three Seasons

#### 3.3.1. Relative Quantification of ApMV and ApNMV during the Spring Season

**Figure 5:**
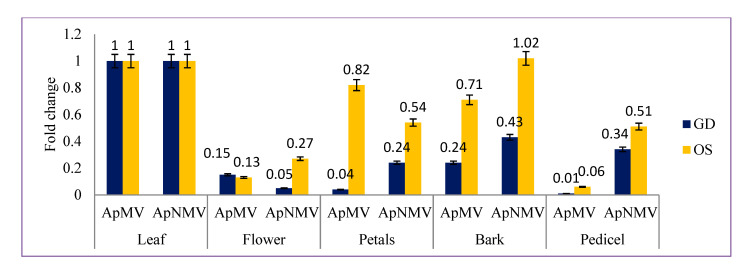
Comparative fold change ofthe CP gene of ApMV and ApNMV in different tissues in cultivar Golden Delicious (GD) and Oregon spur (OS) during the spring season. *Y*-axis represents the fold change in the viral infection, whereas *X*-axis represents different tissues tested for both viruses.

During three seasons (spring, summer and autumn) the relative quantification of ApMV and ApNMV in all tissues in two cultivars, OS and GD, was done using real-time-PCR. Both the viruses were reliably detected in all tested plant tissue samples throughout the year during which the samples were assessed. During the spring season, in cultivar GD, the leaf was the site of maximum expression (1) and the pedicel remained as the site of minimum expression (0.18) with respect to titer in leaf for ApMV. Whereas in cultivar OS, the maximum ApMV titer with respect to leaves was observed in flowers without petals (1.13), whereas the minimum titer was seen in the pedicel (0.6). However, ApNMV showed a different trend as its expression was more in bark for cultivar GD (1.02) and in petals for cultivar OS (0.54), whereas flowers without petals showed the least expression of 0.5 in cultivar GD and 0.27 in cultivar OS. The relative fold change of coat protein gene expression of both viruses in different tissues during the spring season is shown in Figure 5.

#### 3.3.2. Relative Quantification of ApMV and ApNMV during the Summer Season

**Figure 6:**
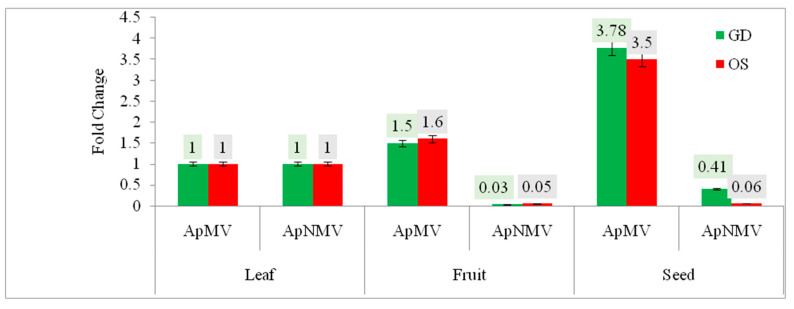
Comparative fold change of the CP gene of ApMV and ApNMV in different tissues in cultivar Golden Delicious (GD) and Oregon spur (OS) during the summer season. *Y*-axis represents the fold change in the virus infection, whereas *X*-axis represents different tissues tested for both viruses.

During the summer season, although the symptoms in the form of a less prominent mosaic pattern were present on leaves in both cultivars (GD and OS), seeds showed maximum ApMV titer (1.0) in both cultivars and leaves displayed minimum titer in both cultivars (0.26 in GD and 0.4 in OS).The ApNMV titer was maximum in leaves in both the GD and OS cultivars (1) and minimum in seed (0.4 for GD and 0.5 for OS).The fold change of coat protein gene expression in both the viruses in three different tissues during the summer season is shown in Figure 6.

#### 3.3.3. Relative Quantification of ApMV and ApNMV during the Autumn Season

**Figure 7:**
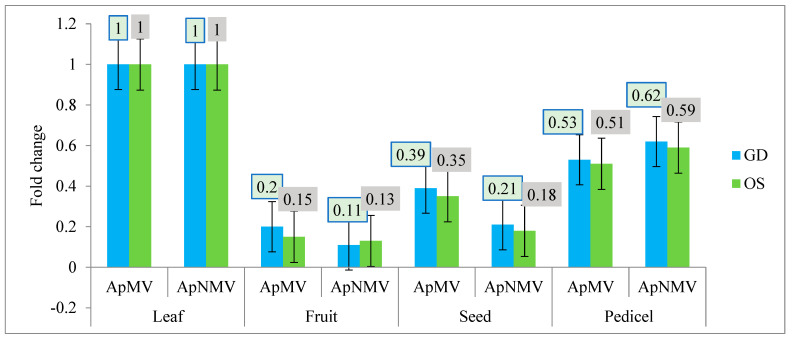
Comparative fold change of the CP gene of APMV and ApNMV in different tissues in cultivar Golden Delicious (GD) and Oregon spur (OS) during the autumn season. *Y*-axis represents the fold change in the virus infection, whereas *X*-axis represents different tissues tested for both viruses.

During the autumn season, the ApMV and ApNMV titer was maximum in leaves (1.0) for both cultivars; however ApMV and ApNMV titer was minimum in bark and seeds (0.2), respectively, for cultivar GD. For cultivar OS, the minimum titer was found in bark (0.15) for ApMV and in seeds (0.19) for ApNMV. The fold change of coat protein gene expression in both viruses in different tissues during the autumn season is shown in Figure 7. The relative expression along with mean Ct values of ApMV and ApNMV in different tissues during the three seasons, viz., spring, summer and autumn is shown in Table 2.

### 3.4. Validation of Optimized Tissue and Time in Various Apple Cultivars

The results obtained for tissue and time optimization based on RT-PCR/qRT-PCR were validated on 7 apple cultivars, i.e., OS, WRJ, GD, SE, GM, JE and A. The results validated the presence of both viruses in tissues standardized during different seasons, as confirmed by RT-PCR/qRT-PCR, as seen above. The presence and absence of viruses along with percent expression of the CP gene is shown in Table 3.

## 4. Discussion

The importance of AMD among viral diseases in apple cultivation is highly significant as it affects crops both in terms of production as well as productivity [28,29]. Both ApMV and ApNMV have been found to be associated with AMD from countries where apples are being grown [13,18]. Thorough understanding of the mechanism of transmission and the dynamics of the virus’ ability to differentially select a particular tissue for faster replication will only be possible if we have a map of the tissues infected by the virus and thus, spatial and temporal study is desired. Also, studying a particular tissue showing a relatively higher titer of virus will help in targeting tissues which can aid in early diagnosis of the virus [30], which in turn will help the apple nursery growers to select a virus-free tissue for grafting and budding. In order to see the spatial and temporal distribution of both viruses (ApMV and ApNMV) for the optimization of tissue and time, the present study was conducted during three different seasons (spring, summer and autumn) with different types of tissues available during each particular season via RT-PCR and qRT-PCR. The DAS-ELISA can also give considerable insights about the presence of virus in different tissues, however, due to the low sensitivity of the technique, results can be false when the titer of the virus is low [24,31,32]. The CP gene is unique to viruses and is, therefore, considered a suitable target for viral detection. The RT-PCR and qRT-PCR not only confirm the presence of viruses with maximal specificity, but qRT-PCR also gives quantitative measurements of the viruses as well [30]. In an attempt to reliably detect the presence of both viruses in various parts of apple plants and optimizes tissue and season for early and round the year detection, different techniques, viz., RT-PCR and qRT-PCR, were used, which proved to be precious tools. In the present study, both RT-PCR and qRT-PCR assays were developed using total genomic RNA extracted from known ApMV/ApNMV-infected plants, maintained in a field gene bank and further validated on seven apple cultivars maintained in the field. The cultivars were selected based on the symptoms of mosaic and necrosis observed in the field and susceptibility to mosaic disease, as reported by earlier studies [8,18]. 

In the spring season, both viruses (ApMV and ApNMV) were detected in all the tissues tested via RT-PCR, however, the expression was greater in leaves for both viruses, as revealed through qRT-PCR. During the summer season, both viruses were not detected in leaves via RT-PCR. Both the viruses were detected through RT-qPCR, but the titer of the ApNMV was lowest in leaves when compared to ApMV. However, during the summer season, the titer of ApMV was highest in seeds when compared to ApNMV. During the autumn season, both viruses were again detected, with leaves having the highest titer. The presence of uneven distribution of both viruses in plant parts is due to the labile nature of these viruses as both viruses belong to the genus *Ilarvirus* [25,29]. The spatial and temporal distribution of both viruses(ApMV and ApNMV) in seven different tissue samples, viz., leaves, flowers without petals, petals, bark, pedicels, fruit and seeds revealed varied presence and expression during all three seasons. The reasons for this could be the difference in temperatures during different seasons and the uneven distribution of both viruses in various tissues [29,33].

These findings were also supported for other apple viruses such as the apple stem grooving virus (ASGV) and apple stem pitting virus (ASPV) [17,34,35]. In summer, the optimum is higher than required for the virus, and seeds, embedded inside the fruit, are at a lower temperature than the leaf, which receives direct sunshine. This may be a plausible reason for the higher infection rate in seeds and fruits compared to leaves in the summer [31].Also, during the spring season, the temperatures are low and sap flow causes the distribution of the virus to different tissues may be fast [30,35]. The change in virus titer during the summer in leaves and other tissues can be correlated with high temperatures as other workers have already confirmed the decrease in titer of ApNMV due to high temperatures [29]; during the summer season, under temperate conditions, the temperatures are very high. The environmental conditions play an important role in determining the titer of the virus, with studies reporting the pathogen being temporarily inactivated by higher temperatures or having a reduced titer high temperatures [36], which may be attributed to RNA gene silencing. Even the temperature difference within the different plant parts/tissues has a significant influence on the virus as there may be an imbalance between viral replication and degradation created by temperature. Higher temperatures than the optimum is detrimental for a virus. The highest detection efficiency was observed in leaves during all seasons, particularly after using qRT-PCR. Results also revealed that cultivar GD tries to resist to some extent as the level of expression was more pronounced in cultivar OS than in GD.

During the spring and autumn seasons, leaves showed the highest detection efficiency for both the viruses, whereas in the summer, seeds remained a dominating zone for ApNMV in both GD and OS. Minimal infection was observed in leaves compared to seeds. Variation in pathogen detection is likely to be influenced by several factors, and these factors could operate simultaneously. Therefore, the reason for the differences in sensitivity is likely complicated. Mitra and Kootstra [37] proposed that one of many causes for detection failures is that woody plants contain many polyphenols and polysaccharides, which can interfere with the sensitivity of viral detection. The inhibitory effects of these compounds might still be present in total nucleic acid extracts [38], which may affect the reverse transcriptase during RT-PCR [39,40]. However, anything that decreases the reliability of conventional RT-PCR is most likely to influence RT-qPCR methods, which are significantly more reliable due to their greater sensitivity [41,42]. The relative quantification of both viruses in different tissues during different seasons will assist in the selection of plant tissue for easy and rapid diagnosis. Also, RT-qPCR is the only method for the detection and quantification of both viruses in the summer season. The importance of the technique increases for proper virus indexing as it can be used for those mother plants which have not come into flowering/bearing and also for bud wood which will be used for budding in the month of August. It is used for mother plants which have not yet flowered and to index bud wood when budding occurs in the month of August; the importance of this technique increases for proper indexing. Hence, the leaf during the spring or autumn is the best detection material for both viruses, whereas in the summer, the immature seed for ApNMV and leaf for ApMV can be used for detection, either via RT-PCR or qRT-PCR.

## 5. Conclusions

This research has aided in the development of a simple quantitative gene expression test for the early detection of ApMV and ApNMV using qRT-PCR. The current study provided insights into the viral load (by real-time measurement) in various tissues, which will aid in the selection of tissues for viral indexing during the certification stage, enabling easier interchange of rootstocks and variations around the world. This research will also assist nursery farmers in indexing their planting material on time, which will eventually aid in the development of clean, virus-free planting material by picking the ideal tissue at the appropriate moment.

## Figures and Tables

**Table 1:** Detection of ApMV and ApNMV in different parts of plants during different seasons in two cultivars through RT-PCR.

S. No.	Tissue	ApMV in Spring	ApNMV in Spring	ApMV in Summer	ApNMV in Summer	ApMV in Autumn	ApNMV in Autumn
1	Leaf	+	+	-	-	+	+
2	Flowers without petals	+	+	NA	NA	NA	NA
3	Petal	+	+	NA	NA	NA	NA
4	Pedicel	+	+	-	-	+	+
5	Bark	+	+	-	-	-	-
6	Fruit	NA	NA	-	-	-	-
7	Seed	NA	NA	+	+	-	-

(+): Detected, (-): Non-detected, NA: Not available during the particular season, Sample size: 3 samples per tissue.

**Table 2:** Relative quantification of ApMV and ApNMV in different tissues of two cultivars of Golden Delicious and Oregon Spur apple cultivars during the spring, summer and autumn seasons.

Tissue	Virus	Average Relative Quantification of CP Gene (Spring Season)		Average Relative Quantification of CP Gene (Summer Season)		Average Relative Quantification of CP Gene (Autumn Season)	
		GD	OS	GD	OS	GD	OS
Leaf	ApMV	100(PC)	100(PC)	25.8	9.18	100(PC)	100(PC)
	ApNMV	100(PC)	100(PC)	26.4	7.4	100(PC)	100(PC)
Flower	ApMV	15.8	113	-	-	-	-
	ApNMV	0.05	27.1	-	-	-	-
Petal	ApMV	4	82	-	-	-	-
	ApNMV	23.9	54.3	-	-	-	-
Bark	ApMV	24.4	71	-	-	2	1.5
	ApNMV	42.6	102	-	-	11.5	10.35
Pedicel	ApMV	1.3	6	-	-	-	-
	ApNMV	34.3	51	-	-	-	-
Fruit	ApMV	-	-	7.9	5.4	53.5	51.4
	ApNMV	-	-	3.0	2.7	92	88.5
Seed	ApMV	-	-	40	44.0	38.9	37.6
	ApNMV	-	-	100(PC)	100(PC)	2	1.95
C.D.		1.25	1.53	1.46	1.04	1.5	1.5
C.V.		2.11	1.26	1.59	1.341	1.7	1.7

PC: Positive calibrator, ApMV: Apple mosaic virus, ApNMV: Apple necrotic mosaic virus, CP: Coat protein, C.D.: Critical difference, C.V.: Coefficient of variation, Sample size: 3 samples per tissue.

**Table 3:** Detection and percent expression of ApMV and ApNMV in different standardized tissues in different seasons in various mosaic-infected cultivars of apple.

	Detection of ApMV Using RT-PCR during Spring Season in Various Cultivars							Detection of ApNMV Using RT-PCR during Spring Season in Various Cultivars						
Tissues	**OS**	**WRJ**	**GD**	**SE**	**GM**	**JE**	**A**	**OS**	**WRJ**	**GD**	**SE**	**GM**	**JE**	**A**
Leaf	+	+	+	+	+	+	+	+	+	+	+	+	+	+
Flower	+	+	+	+	+	+	+	+	+	+	+	+	+	+
Petal	+	+	+	+	+	+	+	+	+	+	+	+	+	+
Pedicel	+	-	+	+	+	+	+	+	+	+	+	+	+	+
Bark	+	+	+	+	+	+	+	+	+	+	+	+	+	+
	**Detection of ApMV Using RT-PCR during Summer Season in Various Cultivars**							**Detection of ApMV Using RT-PCR during Summer Season in Various Cultivars**						
Seed	+	+	+	+	+	+	+	+	+	+	+	+	+	-
	**Detection of ApMV Using RT-PCR during Autumn Season in Various Cultivars**							**Detection of ApMV Using RT-PCR during Autumn Season in Various Cultivars**						
Leaf	+	+	+	+	+	+	+	+	+	+	+	+	+	+
Pedicel	+	+	+	+	+	+	+	+	+	+	+	+	+	+
	**Percent CP Expression of ApMV Using RT-qPCR during Spring Season in Various Cultivars**							**Percent CP Expression of ApNMV Using RT-qPCR during Spring Season in Various Cultivars**						
Tissues	**OS**	**WRJ**	**GD**	**SE**	**GM**	**JE**	**A**	**OS**	**WRJ**	**GD**	**SE**	**GM**	**JE**	**A**
Leaf	100(1)	100(1)	100(1)	100(1)	100(1)	100(1)	100(1)	100(1)	100(1)	100(1)	100(1)	100(1)	100(1)	100(1)
Flower	45.5(0.45)	19.5(0.19)	16.3(0.16)	15.7(0.15)	15.9(0.15)	12.5(0.12)	24.5(0.24)	27.20(0.27)	17.56(0.17)	17.3(0.17)	19.7(0.2)	12.9(0.2)	12.5(0.1)	16.5(0.16)
Petal	15.5(0.15)	11.6(0.11)	10.3(0.1)	11.1(0.11)	7.1(0.07)	9.50(0.09)	10.4(0.10)	17.20(0.17)	10.60(0.10)	11.3(0.11)	16.2(0.16)	7.19(0.07)	9.50(0.09)	10.5(0.1)
Pedicel	35.2(0.35)	23.5(0.23)	23.2(0.2)	35.7(0.35)	31.9(0.31)	31.5(0.31)	39.9(0.39)	32.81(0.32)	27.43(0.27)	33.2(0.33)	31.45(0.3)	37.23(0.37)	35.3(0.35)	40.50(0.4)
Bark	12.3(0.12)	10.5(0.10)	6.4(0.06)	5.71(0.57)	9.7(0.09)	11.7(0.11)	13.3(0.13)	11.78(0.12)	11.76(0.12)	6.4(0.06)	7.87(0.07)	7.72(0.07)	10.73(0.1)	12.13(0.1)
	**Percent CP Expression of ApMV Using RT-qPCR during Summer Season in Various Cultivars**							**Percent CP Expression of ApNMV Using RT-qPCR during Summer Season in Various Cultivars**						
Seed	100(1)	100(1)	100(1)	100(1)	100(1)	100(1)	100(1)	100(1)	100(1)	100(1)	100(1)	100(1)	100(1)	100(1)
	**Percent CP Expression of ApMV Using RT-qPCR during Autumn Season in Various Cultivars**							**Percent CP Expression of ApNMV Using RT-qPCR during Autumn Season in Various Cultivars**						
Leaf	100(1)	100(1)	100(1)	100(1)	100(1)	100(1)	100(1)	100(1)	100(1)	100(1)	100(1)	100(1)	100(1)	100(1)
Pedicel	20.10(0.2)	23.5(0.23)	22.8(0.2)	19.8(0.2)	21.5(0.2)	18.9(0.19)	18.8(0.19)	26.10(0.26)	29.6(0.3)	27.8(0.28)	29.8(0.3)	22.5	28.9(0.29)	25.8(0.26)

OS, Welson Red June—WRJ, GD, Sharp Earliest—SE, Gala Mast—GM, June Eating—JE and Ambri—A, (+): Presence, (-): Absence, the values in brackets represent the fold change of CP gene expression, Sample size: 3 samples per tissue.

## Data Availability

All the data is available with corresponding author which will be made available on request.
